# Ranking differentially expressed genes from Affymetrix gene expression data: methods with reproducibility, sensitivity, and specificity

**DOI:** 10.1186/1748-7188-4-7

**Published:** 2009-04-22

**Authors:** Koji Kadota, Yuji Nakai, Kentaro Shimizu

**Affiliations:** 1 Graduate School of Agricultural and Life Sciences, The University of Tokyo, 1-1-1 Yayoi, Bunkyo-ku, Tokyo 113-8657, Japan

## Abstract

**Background:**

To identify differentially expressed genes (DEGs) from microarray data, users of the Affymetrix GeneChip system need to select both a preprocessing algorithm to obtain expression-level measurements and a way of ranking genes to obtain the most plausible candidates. We recently recommended suitable combinations of a preprocessing algorithm and gene ranking method that can be used to identify DEGs with a higher level of sensitivity and specificity. However, in addition to these recommendations, researchers also want to know which combinations enhance reproducibility.

**Results:**

We compared eight conventional methods for ranking genes: weighted average difference (WAD), average difference (AD), fold change (FC), rank products (RP), moderated *t *statistic (modT), significance analysis of microarrays (samT), shrinkage *t *statistic (shrinkT), and intensity-based moderated *t *statistic (ibmT) with six preprocessing algorithms (PLIER, VSN, FARMS, multi-mgMOS (mmgMOS), MBEI, and GCRMA). A total of 36 real experimental datasets was evaluated on the basis of the area under the receiver operating characteristic curve (AUC) as a measure for both sensitivity and specificity. We found that the RP method performed well for VSN-, FARMS-, MBEI-, and GCRMA-preprocessed data, and the WAD method performed well for mmgMOS-preprocessed data. Our analysis of the MicroArray Quality Control (MAQC) project's datasets showed that the FC-based gene ranking methods (WAD, AD, FC, and RP) had a higher level of reproducibility: The percentages of overlapping genes (POGs) across different sites for the FC-based methods were higher overall than those for the *t*-statistic-based methods (modT, samT, shrinkT, and ibmT). In particular, POG values for WAD were the highest overall among the FC-based methods irrespective of the choice of preprocessing algorithm.

**Conclusion:**

Our results demonstrate that to increase sensitivity, specificity, and reproducibility in microarray analyses, we need to select suitable combinations of preprocessing algorithms and gene ranking methods. We recommend the use of FC-based methods, in particular RP or WAD.

## Background

Microarray analysis is often used to detect differentially expressed genes (DEGs) under different conditions. As there are considerable differences [1,2] in how well it performs, choosing the best method of ranking these genes is important. Furthermore, Affymetrix GeneChip users need to choose a preprocessing algorithm from a number of competitors in order to obtain expression-level measurements [3].

We recently reported with another group that there are suitable combinations of preprocessing algorithms and gene ranking methods [1,2]. We evaluated three preprocessing algorithms, MAS [4], RMA [5], and DFW [6], and eight gene ranking methods, WAD [1], AD, FC, RP [7], modT [8], samT [9], shrinkT [10], and ibmT [11], by using a total of 38 datasets (including 36 real experimental datasets) [1]. Meanwhile, Pearson [2] evaluated nine preprocessing algorithms, MAS [4], RMA [5], DFW [6], MBEI [12], CP [13], PLIER[14], GCRMA [15], mmgMOS [16], and FARMS [17], and five gene ranking methods, modT [8], FC, a standard *t*-test, cyberT [18], and PPLR [19], by using only one artificial 'spike-in' dataset, the Golden Spike dataset [13].

When we re-evaluated the two reports using the common algorithms and methods we found that suitable gene ranking methods for each of the three preprocessing algorithms, i.e., MAS, RMA, and DFW, converge to the same: Combinations of MAS and modT (MAS/modT), RMA/FC, and DFW/FC can thus be recommended. However, the final conclusions for the original reports are understandably different: Our recommendations [1] are MAS/WAD, RMA/FC, and DFW/RP, while Pearson [2] recommends mmgMOS/PPLR, GCRMA/FC, and so on. This difference is mainly because fewer preprocessing algorithms were evaluated in our previous study [1].

We investigated suitable gene ranking methods for each of six preprocessing algorithms: MBEI, VSN [20], PLIER, GCRMA, FARMS, and mmgMOS. We also investigated the best combination of a preprocessing algorithm and gene ranking method using another evaluation metric, i.e., the percentage of overlapping genes (POG), proposed by the MAQC study [21].

Most authors of methodological papers have made claims that their methods have a greater area under the receiver operating characteristic curve (AUC) values, i.e., both high sensitivity and specificity [1,2]. However, reproducibility is rarely mentioned [21]. A good method should produce high POG values, i.e., those indicating reproducibility as well as high AUC ones, i.e., those for sensitivity and specificity. We will discuss suitable combinations of preprocessing algorithms and gene ranking methods.

## Results and discussion

### Evaluation based on AUC metric

Our reason for using this evaluation was to investigate suitable gene ranking methods for each preprocessing algorithm. Six algorithms were investigated: Three that have sometimes been used (Table 1), MBEI (PM only model) [12], GCRMA [15], and VSN [20], one that was used by the MAQC study [21], PLIER [14], one that was the best method in a benchmark study [22] at the time of writing, FARMS [17], and one recommended by Pearson [2,23], mmgMOS [16].

**Table 1:** Frequency of preprocessing algorithms used during 2003 – 2008

	2003	2004	2005	2006	2007	2008
MAS (2002)	8	34	53	42	47	16
RMA (2003)		8	15	29	20	9
MBEI (2001)	0	3	7	16	8	3
GCRMA (2004)			0	5	8	4
VSN (2002)	0	0	0	4	0	2

Our investigation was performed for 394 different papers with analyses performed using the Affymetrix HG-U133A array (Gene Expression Omnibus (GEO) ID: GPL96) [32]. These results were obtained by reading the entire texts. Statistics for top five algorithms used are shown, and years in parentheses refer to their publication. Suitable methods of ranking genes for the first two algorithms, MAS and RMA, have been discussed [1].

The AUC enables comparison based on sensitivity and specificity simultaneously because the ROC curve is created by plotting the true positive (TP) rate (sensitivity) against the false positive (FP) rate (1 minus the specificity) obtained at each possible threshold value [1]. A good method should produce high AUC values for real experimental datasets [1]. We evaluated 36 real experimental datasets, each of which has some true DEGs confirmed by a real-time polymerase chain reaction, RT-PCR. They correspond to "Datasets 3–38" in our previous study (for details, see reference [1]). The 36 experimental datasets can be divided into two groups: One group (Datasets 3–26) had originally been analyzed using MAS-preprocessed data and the other (Datasets 27–38) with RMA-preprocessed data. We will use these serial numbers, i.e., Datasets 3–38, for the 36 datasets. We do not use the first two artificial spike-in datasets (Datasets 1 and 2) here. This is because the results for the real experimental datasets should take precedence over those for the artificial ones [1].

The average AUC values for PLIER-, VSN-, FARMS-, mmgMOS-, MBEI-, and GCRMA-preprocessed data for the two groups, Datasets 3–26 and Datasets 27–38, are shown in Table 2. The values for each dataset are given in the additional file [see Additional file 1]. To make this work comparable with our previous results for MAS-, RMA-, and DFW-preprocessed data (table four in [1]), the average AUC values are also provided. The values for the mmgMOS- and VSN-preprocessed data for the first and second groups were the best overall among the six preprocessing algorithms respectively. The high AUC values for the mmgMOS-preprocessed data for the first group can be explained by the similarity between the mmgMOS- and MAS-preprocessed data (Figure 1 in [3]). The high values for the VSN- and GCRMA-preprocessed data for the second group are also reasonable because these algorithms are quite similar to RMA, which was used in the original papers for the second group (Datasets 27–38). There is thus no convincing rationale for choosing among different preprocessing algorithms with the results from the 36 real experimental datasets that were first analyzed using only MAS- or RMA-preprocessed data. Therefore, we will now discuss suitable gene ranking methods for each preprocessing algorithm.

**Figure 1:**
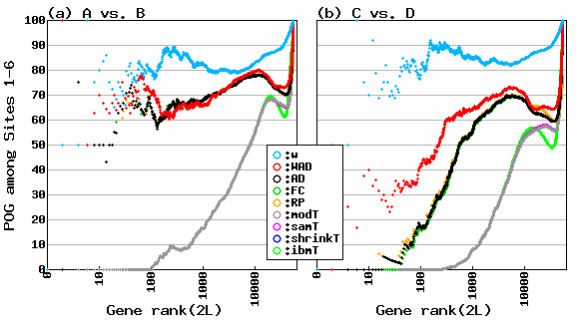
**POG values for FARMS-preprocessed data**. (a) Sample A vs. Sample B; (b) Sample C vs. Sample D. Number of DEGs is shown on *x*-axis and *L *is the number of DEGs from the up-regulation in one sample (Sample A or C) or other sample (Sample B or D) [21]. Percentage of genes (POG) common to the six gene lists derived from six test sites at a given number of DEGs is shown on *y*-axis. Note that POG values for shrinkT were not shown because statistics frequently include infinities. Also, that the reproducibility (POG) shown by only the *w *term (light blue line) in WAD is clearly higher than that shown by the AD (black line) recommended by the MAQC study.

**Table 2:** Average AUC values for Datasets 3–26 and 27–38

Method	PLIER	VSN	FARMS	mmgMOS	MBEI	GCRMA	MAS	RMA	DFW	Average
Datasets 3–26										
WAD	89.15	90.97	91.58	**94.84**	88.19	92.67	**96.74**	91.37	91.41	91.88
AD	87.32	**92.96**	92.47	93.13	89.14	**93.42**	93.76	93.10	92.24	91.95
FC	86.20	92.92	92.49	92.82	89.71	93.16	93.63	**93.12**	92.24	91.81
RP	**92.01**	92.48	**93.06**	92.20	**90.07**	93.23	91.51	92.54	**92.53**	**92.18**
modT	85.43	90.70	89.23	91.91	86.79	91.19	95.67	91.38	90.11	90.27
samT	85.25	90.94	89.40	92.99	87.09	91.07	95.95	91.23	89.96	90.43
shrinkT	84.65	90.62	-	92.56	86.89	92.12	95.73	91.32	91.45	90.67
ibmT	86.27	91.02	90.04	93.11	87.10	91.06	96.34	91.77	90.25	90.78
Datasets 27–38										
WAD	91.30	95.41	93.55	**92.36**	93.39	95.75	**92.42**	96.73	94.09	**93.89**
AD	89.01	96.22	93.81	86.99	93.11	96.18	87.41	**96.77**	94.22	92.64
FC	88.82	96.20	93.81	85.92	92.94	96.06	88.23	96.73	94.22	92.55
RP	**91.57**	**96.53**	**94.76**	86.94	**93.53**	**96.25**	84.55	96.53	**94.67**	92.81
modT	89.28	94.53	91.62	89.61	90.50	93.33	90.90	95.28	92.36	91.93
samT	88.53	95.08	91.90	89.14	90.49	93.60	90.31	95.70	92.05	91.87
shrinkT	88.56	94.04	-	89.85	90.13	94.48	90.97	94.85	93.68	92.07
ibmT	89.89	94.80	89.95	90.60	90.66	93.67	91.92	95.49	92.43	92.16
Average	88.33	93.46	91.98	90.94	89.98	93.58	92.25	93.99	92.37	

As statistics for shrinkT frequently include infinities, AUC values for FARMS/shrinkT could not be calculated. Note that AUC values for each dataset are given in the additional file [see Additional file 1].

The best gene ranking methods for the four (PLIER, FARMS, mmgMOS, and MBEI) of the six preprocessing algorithms were the same for both groups: PLIER/RP, FARMS/RP, mmgMOS/WAD, and MBEI/RP showed the highest average AUC values (Table 2). For VSN- and GCRMA-preprocessed data, AD and RP were the best for the first and second groups respectively. In practice, both gene ranking methods for the two preprocessing algorithms can be selected because the average AUC values for VSN/RP and GCRMA/RP for the first group and the values for VSN/AD and GCRMA/AD for the second group are close to those for the corresponding best combinations.

A recent study [2] reported that the use of the probability of positive log ratio (PPLR) method [19] combined with mmgMOS was the best when another spike-in dataset was evaluated using AUC. We evaluated the performance of the PPLR method combined with only the mmgMOS-preprocessed data because the gene ranking method was originally designed for the mmgMOS-preprocessed data and because the method may take a long time to run. The average AUC values for mmgMOS/PPLR were high (93.34 for the first and 90.06 for the second) but not the best ones. Of course, there might be the other good preprocessing algorithms with good affinity with PPLR and evaluating them will be our next task.

It should be noted that the average AUC values listed in Table 2 were obtained using data in which log signals under 0 were set to 0 though two preprocessing algorithms (PLIER and mmgMOS) are designed to output both positive and negative log signals. We observed that the best average AUC values for PLIER- and mmgMOS-preprocessed data with no floor value setting for the two groups, Datasets 3–26 and Datasets 27–38, were lower than those corresponding values obtained from log signal data with the floor value setting (data not shown). This result suggests that using a floor value setting can increase the sensitivity and specificity with which DEGs are detected.

### Evaluation based on POG metric

The authors of the MAQC study have developed a large number of reference datasets to address the reproducibility of microarray results [21]. In the study for Affymetrix GeneChip data, the MAQC study compared five gene ranking methods: AD, samT, a simple *t*-statistic, the Wilcox rank-sum test, and random, and they concluded that a FC-based method (AD) showed the most reproducible result when intra-platform reproducibilities for DEGs, i.e., the percentage of overlapping genes (POG), among test sites were evaluated. However, the evaluation was performed only for the PLIER-preprocessed data: The conclusion of the MAQC project is not necessarily universal when the other preprocessing algorithms are employed. Therefore, researchers would like to compare many gene ranking methods with other preprocessing algorithms using POG as an evaluation metric.

The authors of the above study used two RNA sample types and two mixtures of the original samples: Sample A, a universal human reference RNA; Sample B, a human brain reference RNA; Sample C, which consisted of 75 and 25% of sample A and B respectively; and Sample D, which consisted of 25 and 75% of sample A and B respectively. At each of the test sites, five replicate assays for each of the four sample types were performed. Since the Affymetrix GeneChip data were generated at six test sites, the evaluation was performed using the POG values among six test sites.

The POG values for the 100 top-ranked genes among six test sites are shown in Table 3. Recall that the MAQC study reports that a FC-based gene ranking (AD) generates more reproducible results compared to those gained from a *t*-statistic-based gene ranking such as samT [9] when the POG metric is used [21]. Although the first study was performed only for the PLIER-preprocessed data, this tendency was universally observed for the other preprocessed data: FC-based methods are superior to *t*-statistic-based methods if the POG is evaluated.

**Table 3:** POG values for the 100 top-ranked genes among six test sites

Method	PLIER	VSN	FARMS	mmgMOS	MBEI	GCRMA	MAS	RMA	DFW
Sample A vs. B									
WAD	64	56	**71**	65	69	58	61	62	60
AD	45	59	65	62	58	65	45	64	59
FC	40	60	65	60	58	65	45	64	59
RP	43	58	65	65	59	65	42	63	59
modT	1	22	0	3	15	5	6	1	1
samT	11	11	0	55	39	6	31	10	1
shrinkT	0	12	-	37	20	10	5	4	1
ibmT	-	21	0	-	15	15	8	3	1
Sample C vs. D									
WAD	8	36	**41**	12	33	9	8	20	10
AD	1	6	17	13	4	4	1	4	4
FC	1	8	16	14	2	3	0	5	4
RP	2	10	17	12	4	3	1	5	4
modT	0	0	0	0	0	0	2	1	0
samT	2	0	0	13	1	0	3	3	0
shrinkT	0	0	-	0	2	0	0	1	0
ibmT	-	0	0	-	1	1	-	6	0

Various POG values such as for FARMS/shrinkT could not be calculated because those statistics include infinities. Note POG values for MAS, RMA, and DFW-preprocessed data.

The best combination was FARMS/WAD. When compared to the MAQC's recommendation, the WAD was clearly superior to the AD method. This is because WAD consists of AD statistics and a weight term *w *[1] and the gene ranking based on the *w *statistic is much more reproducible than the one based on the AD statistic. The reality for *w *is relative average signal intensity on log-scale so that highly expressed genes are highly ranked on the average for the different conditions [1,24]. A representative example of the reproducibility for the *w *term in WAD is shown in Figure 1. The POG values among six test sites for the *w *statistic (blue line) are clearly higher than those for the AD statistic (black line). We confirmed this tendency for all gene expression data preprocessed using a total of nine algorithms listed in Table 3 (see Additional file 2).

If we follow only the MAQC's evaluation metric, the most reproducible gene ranking method should be the *w *statistic (not the WAD one). However, the *w *statistic merely indicates relative average signal intensity [1] and this statistic is clearly inferior to that obtained from the other specialized gene ranking methods such as WAD when AUC value (both sensitivity and specificity) is evaluated (see Additional file 1).

### Choice of best gene ranking methods with both high AUC and high POG values

Note that the authors of the MAQC study recommend the use of both the AD and a *t*-statistic-based method with a non-stringent *p *threshold. This is despite the fact that only using the AD method can increase the reproducibility [21]. The reason for this recommendation was found in a recent study [25] that explained how using *t*-statistic-based methods allowed specificity and sensitivity to be controlled.

Our current and previous [1] results are clearly different from that of MAQC's recommendation. Our recommendation is that FC-based methods should be used for increasing both the POG values i.e., reproducibility and the AUC values i.e., sensitivity and specificity. Specifically, the recommendations can be narrowed to RP or WAD. When the RP method is combined with several preprocessing algorithms, PLIER, VSN, FARMS, MBEI, and GCRMA, the AUC value is enhanced. However, as shown in Tables 2 and 3, when WAD is combined with mmgMOS this enhances the AUC values and when WAD with all preprocessing algorithms is interrogated overall it enhances the POG values.

The difference between the recommendations of the MAQC study and of ours is mainly because the two methods we recommended, RP and WAD, are not included in the MAQC study. This is understandable because there are many other strategies, such as those described in [26-28], that we did not analyze here. This implies the use of RP or WAD may not be the best; however, we maintain that our recommendations are a better choice compared to that of the MAQC study [21,25]. Since new preprocessing algorithms and gene ranking methods continue to be developed [29-31], the search for the best combination for identifying the true DEGs is ongoing.

## Conclusion

We evaluated the performance of combinations between six preprocessing algorithms and eight gene ranking methods in terms of the AUC value, i.e., both sensitivity and specificity, and the POG one, i.e., reproducibility. Our comprehensive evaluation confirmed the importance of using suitable combinations of preprocessing algorithms and gene ranking methods.

Overall, two FC-based gene ranking methods (RP and WAD) can be recommended. Our current and previous results indicate that any of the following combinations, RMA/RP, DFW/RP, PLIER/RP, VSN/RP, FARMS/RP, MBEI/RP, GCRMA/RP, MAS/WAD, and mmgMOS/WAD, enhances both sensitivity and specificity, and also that using the WAD method enhances reproducibility.

## Methods

The raw data (Affymetrix CEL files) for Datasets 3–38 were obtained from the Gene Expression Omnibus (GEO) website [32]. All analysis was performed using R (ver. 2.7.2) [33] and Bioconductor [34]. The versions of R libraries used in this study are as follows: *plier *(ver. 1.10.0), *vsn *(3.2.1), *farms *(1.3), *puma *(1.6.0), *affy *(1.16.0) [35], *gcrma *(2.10.0), *RankProd *(2.12.0) [36], *st *(1.0.3) [10], *limma *(2.14.7) [8], *ROC *(1.14.0). The main functions in the R libraries are as follows: *justPlier *for PLIER, *vsnrma *for VSN, *q.farms *for FARMS, *mmgmos *for mmgMOS, *expresso *for MBEI (PM only model), *gcrma *for GCRMA, *mas5 *for MAS, *rma *for RMA, *expresso *and the R codes available in [37] for DFW, *RP *for RP, *modt.stat *for modT, *sam.stat *for samT, *shrinkt.stat *for shrinkT, *IBMT *for ibmT [38], and *pumaComb *and *pumaDE *for PPLR [19].

Since the MBEI and MAS expression measures do not output logged values, signal intensities under 1 in those preprocessed data were set to 1 so that the logarithm of the data could be found. Logged values smaller than 0 in PLIER-, VSN-, FARMS-, mmgMOS-, and GCRMA-preprocessed data were set to 0. For reproducible research, we made the R code for analyzing Dataset 4 (GEO ID: GSM189708–189713) available as the additional file [see Additional file 3]. The R codes for the other datasets are available upon request.

The raw data for the MAQC datasets were obtained from the MAQC website [39]. The evaluation based on POG was done with 12 datasets produced by the MAQC project [21] in which two RNA sample types and two mixtures of the original samples were used: Sample A, a universal human reference RNA; Sample B, a human brain reference RNA; Sample C, which consisted of 75 and 25% of Sample A and B respectively; and Sample D, which consisted of 25 and 75% of Sample A and B respectively. Five replicate experiments for each of the four sample types at six independent test sites (Sites 1–6) were conducted, and, thus there are 20 files at each site. The data preprocessing was performed at each site. The application of the gene ranking methods was independently performed for comparisons of "Sample A versus B" and "Sample C versus D".

## Abbreviations

AUC: area under ROC curve; CP: Choe's preferred (method); DEG: differentially expressed gene; DFW: distribution-free weighted (method); FARMS: factor analysis for robust microarray summarization; FC: fold change; FP: false positive; ibmT: intensity-based moderated *t*-statistic; MAQC: microarray quality control (project); MAS: (Affymetrix) MicroArray Suite version 5; MBEI: model-based expression index; mmgMOS: multi-mgMOS; modT: moderated *t*-statistic; PLIER: probe logarithmic intensity error; POG: percentage of overlapping genes; PPLR: probability of positive log ratio; RMA: robust multi-chip average; ROC: receiver operating characteristic; RP: rank products; samT: significance analysis of microarrays; shrinkT: shrinkage *t*-statistic; TP: true positive; VSN: variance stabilization; WAD: weighted average difference (method).

## Competing interests

The authors declare that they have no competing interests.

## Authors' contributions

KK performed analysis and wrote the paper. YN performed analysis. KS provided critical comments and led the project.

## Supplementary Material

Additional File 1 **Detailed information for Datasets 3–38.** Click here for file

Additional File 2 **POG values for PLIER-, VSN-, mmgMOS-, MBEI-, GCRMA-, MAS-, RMA-, and DFW-preprocessed data.** Legends are the same as given in Figure 1. Click here for file

Additional File 3 **R-code for analyzing Dataset 4.** Click here for file
