# Supplementary material for: Ranking differentially expressed genes from Affymetrix gene expression data: methods with reproducibility, sensitivity, and specificity
Source: Algorithms Mol Biol. 2009 Apr 22;4:7. doi: 10.1186/1748-7188-4-7 (PMC2679019; doi:10.1186/1748-7188-4-7)
Supplement: Additional File 1 — Detailed information for Datasets 3–38. [file 1748-7188-4-7-S1.doc]

**Detailed information for Datasets 3-38**

**Dataset serial number**

**1. PubMed ID for the original paper:**

**2. Gene Expression Omnibus (GEO) series ID:**

**3. GEO sample IDs in one state:**

**4. GEO sample IDs in the other state:**

**5. Number of probesets and the IDs confirmed by RT-PCR:**

**6. Preprocessing algorithm used in the original study:**

**7. Preprocessing in this study:**

**8. Results (Percent AUC values)**

**Dataset 3**

**1.** http://www.ncbi.nlm.nih.gov/sites/entrez?Db=Pubmed&term=15728662[UID]

**2.** GSE1462

**3.** 3 normal subjects:

GSM24652-24654

**4.** 4 mtDNA “Common”-deletion subjects:

GSM24663-24666

**5.** 4 probesets.

"204388_s_at", "204173_at", "204570_at", "212160_at"

**6.** MAS5.0

**7.** Preprocessed data were obtained from a total of **15** samples in GSE1462.

**8.**

| Method | PLIER | VSN | FARMS | mmgMOS | MBEI | GCRMA |
| --- | --- | --- | --- | --- | --- | --- |
| *w* | 79.246% | 79.058% | 76.979% | 79.466% | 75.623% | 87.653% |
| WAD | 84.305% | 98.685% | 97.304% | 99.225% | 86.483% | 94.912% |
| AD | 76.029% | 95.895% | 97.417% | 91.806% | 78.378% | 91.160% |
| FC | 71.347% | 95.191% | 97.409% | 90.675% | 78.371% | 90.708% |
| RP | 91.347% | 97.062% | 96.814% | 88.980% | 75.621% | 93.477% |
| modT | 55.326% | 85.794% | 68.493% | 87.069% | 76.293% | 93.980% |
| samT | 56.714% | 90.167% | 69.063% | 90.997% | 77.670% | 93.899% |
| shrinkT | 50.985% | 84.714% | 10.206% | 89.354% | 74.792% | 93.790% |
| ibmT | 62.236% | 84.923% | 70.142% | 92.383% | 76.875% | 92.142% |

**Dataset 4**

**1.** http://www.ncbi.nlm.nih.gov/sites/entrez?Db=Pubmed&term=17555786[UID]

**2.** GSE7819

**3.** 3 SVG-A:

GSM189708-189710

**4.** 3 SVGR2:

GSM189711-189713

**5.** 11 probesets.

"212154_at", "212157_at", "212158_at", "205207_at", "209239_at",

"208200_at", "210118_s_at", "205798_at", "210073_at", "202859_x_at",

"211506_s_at"

**6.** MAS5.0

**7.** Preprocessed data were obtained from a total of 6 samples in GSE7819.

**8.**

| Method | PLIER | VSN | FARMS | mmgMOS | MBEI | GCRMA |
| --- | --- | --- | --- | --- | --- | --- |
| *w* | 57.736% | 62.200% | 59.487% | 60.741% | 59.638% | 64.860% |
| WAD | 93.685% | 86.782% | 88.758% | 97.079% | 82.216% | 81.970% |
| AD | 94.850% | 92.126% | 90.361% | 96.027% | 85.176% | 82.850% |
| FC | 94.307% | 92.207% | 90.410% | 95.279% | 85.061% | 83.711% |
| RP | 92.772% | 88.683% | 87.364% | 96.187% | 85.411% | 85.561% |
| modT | 94.850% | 91.424% | 80.546% | 94.591% | 85.727% | 80.062% |
| samT | 94.200% | 90.793% | 79.957% | 96.675% | 85.795% | 79.967% |
| shrinkT | 94.207% | 90.966% | 85.377% | 96.327% | 85.724% | 80.514% |
| ibmT | 95.026% | 92.812% | 83.709% | 96.844% | 86.968% | 81.471% |

**Dataset 5**

**1.** http://www.ncbi.nlm.nih.gov/sites/entrez?Db=Pubmed&term=17490972[UID]

**2.** GSE8441

**3.** 11 control groups:

GSM209811, GSM209813, GSM209815, GSM209817, GSM209819,

GSM209821, GSM209823, GSM209825, GSM209827, GSM209829,

GSM209831

**4.** 11 inadequate dietary protein intake groups:

GSM209812, GSM209814, GSM209816, GSM209818, GSM209820,

GSM209822, GSM209824, GSM209826, GSM209828, GSM209830,

GSM209832

**5.** 9 probesets.

"201721_s_at", "221232_s_at", "211025_x_at", "211699_x_at", "204802_at",

"208581_x_at", "202310_s_at", "204051_s_at", "215049_x_at"

**6.** MAS5.0

**7.** Preprocessed data were obtained from a total of 22 samples in GSE8441.

**8.**

| Method | PLIER | VSN | FARMS | mmgMOS | MBEI | GCRMA |
| --- | --- | --- | --- | --- | --- | --- |
| *w* | 86.742% | 84.436% | 84.113% | 87.130% | 83.480% | 80.315% |
| WAD | 99.750% | 98.391% | 98.716% | 99.681% | 98.418% | 98.491% |
| AD | 98.088% | 99.624% | 98.912% | 95.222% | 99.378% | 99.694% |
| FC | 98.530% | 99.666% | 98.830% | 92.503% | 99.212% | 99.152% |
| RP | 96.627% | 99.434% | 99.413% | 91.299% | 98.785% | 99.091% |
| modT | 95.725% | 93.065% | 90.639% | 93.170% | 92.400% | 92.766% |
| samT | 96.371% | 95.184% | 91.319% | 94.985% | 94.390% | 94.440% |
| shrinkT | 95.488% | 92.661% | 10.533% | 94.460% | 93.342% | 94.286% |
| ibmT | 97.236% | 93.993% | 91.123% | 94.264% | 93.077% | 93.194% |

**Dataset 6**

**1.** http://www.ncbi.nlm.nih.gov/sites/entrez?Db=Pubmed&term=18029387[UID]

**2.** GSE9499

**3.** 15 normal cases: GSM241000-GSM241014

**4.** 7 ICF syndrome cases: GSM241015-GSM241021

**5.**  77 probesets.

"215356_at", "213194_at", "204201_s_at", "205403_at", "211372_s_at",

"201122_x_at", "201123_s_at", "204866_at", "207426_s_at", "204072_s_at",

"209582_s_at", "209583_s_at", "201565_s_at", "206337_at", "206140_at",

"211219_s_at", "206571_s_at", "218181_s_at", "204897_at", "204689_at",

"215933_s_at", "205991_s_at", "212464_s_at", "211719_x_at", "216442_x_at",

"210495_x_at", "177_at", "215723_s_at", "201876_at", "210830_s_at",

"218229_s_at", "220643_s_at", "206489_s_at", "206490_at", "205039_s_at",

"221773_at", "221210_s_at", "202674_s_at", "220188_at", "213103_at",

"210629_x_at", "215633_x_at", "214574_x_at", "205290_s_at", "201349_at",

"206150_at", "204445_s_at", "204446_s_at", "214366_s_at", "206513_at",

"209771_x_at", "216379_x_at", "266_s_at", "205124_at", "213236_at",

"41644_at", "209079_x_at", "205717_x_at", "211066_x_at", "205098_at",

"205099_s_at", "204890_s_at", "204891_s_at", "209569_x_at", "211577_s_at",

"33304_at", "204698_at", "217983_s_at", "217984_at", "209269_s_at",

"207540_s_at", "205484_at", "213664_at", "204159_at", "210992_x_at",

"211395_x_at", "211644_x_at"

**6.** MAS5.0

**7.** Preprocessed data were obtained from a total of 22 samples in GSE9499.

**8.**

| Method | PLIER | VSN | FARMS | mmgMOS | MBEI | GCRMA |
| --- | --- | --- | --- | --- | --- | --- |
| *w* | 56.780% | 56.695% | 57.164% | 57.516% | 54.553% | 59.307% |
| WAD | 97.218% | 91.859% | 93.372% | 98.335% | 92.620% | 93.194% |
| AD | 96.955% | 96.882% | 94.478% | 98.741% | 96.039% | 95.629% |
| FC | 97.147% | 96.820% | 94.433% | 98.407% | 96.017% | 95.588% |
| RP | 97.131% | 97.029% | 95.765% | 98.765% | 96.191% | 95.446% |
| modT | 97.686% | 98.062% | 96.005% | 98.741% | 96.756% | 96.177% |
| samT | 97.677% | 98.098% | 96.107% | 99.211% | 96.838% | 96.227% |
| shrinkT | 97.677% | 98.058% | 20.144% | 98.949% | 96.720% | 96.258% |
| ibmT | 97.712% | 98.125% | 96.126% | 98.893% | 96.788% | 96.303% |

**Dataset 7**

**1.** http://www.ncbi.nlm.nih.gov/sites/entrez?Db=Pubmed&term=14872006[UID]

**2.** GSE974

**3.** 19 pre:

GSM14844, GSM14938, GSM14940, GSM14942, GSM14944,

GSM14946, GSM14948, GSM14950, GSM14952, GSM14954,

GSM14956, GSM14958, GSM14960, GSM14962, GSM14965,

GSM14967, GSM14969, GSM14971, GSM14973

**4.** 19 post:

GSM14936, GSM14937, GSM14939, GSM14941, GSM14943,

GSM14945, GSM14947, GSM14949, GSM14951, GSM14953,

GSM14955, GSM14957, GSM14959, GSM14961, GSM14963,

GSM14966, GSM14968, GSM14970, GSM14972

**5.** 3 probesets.

"212558_at", "212298_at", "205517_at"

**6.** MAS5.0

**7.** Preprocessed data were obtained from a total of 38 samples in GSE974.

**8.**

| Method | PLIER | VSN | FARMS | mmgMOS | MBEI | GCRMA |
| --- | --- | --- | --- | --- | --- | --- |
| *w* | 81.429% | 82.831% | 81.399% | 85.525% | 83.508% | 87.986% |
| WAD | 94.660% | 95.865% | 81.412% | 92.977% | 81.679% | 96.770% |
| AD | 91.924% | 96.505% | 80.265% | 84.451% | 75.299% | 95.060% |
| FC | 89.019% | 96.133% | 80.266% | 87.980% | 91.673% | 92.422% |
| RP | 94.343% | 97.513% | 85.655% | 79.620% | 95.196% | 97.313% |
| modT | 85.393% | 91.643% | 96.830% | 83.019% | 70.194% | 88.223% |
| samT | 85.712% | 93.999% | 96.984% | 82.720% | 70.687% | 88.884% |
| shrinkT | 85.266% | 92.338% | 45.045% | 83.471% | 70.488% | 90.627% |
| ibmT | 85.476% | 91.631% | 96.952% | 83.419% | 70.157% | 88.238% |

**Dataset 8**

**1.** http://www.ncbi.nlm.nih.gov/sites/entrez?Db=Pubmed&term=16617158[UID]

**2.** GSE2638 and GSE2639

**3.** 7 control samples:

GSM50771-50773 and GSM50777-50780

**4.** 7 TNF-stimulated samples:

GSM50774-50776 and GSM50781-50784

**5.** 13 probesets.

"211506_s_at", "216598_s_at", "214974_x_at", "204470_at", "210511_s_at",

"205992_s_at", "209716_at", "207850_at", "205476_at", "205798_at",

"207196_s_at", "212641_at", "212642_s_at"

**6.** MAS5.0

**7.** Preprocessed data were obtained from a total of 14 samples in GSE2638 and GSE2639.

**8.**

| Method | PLIER | VSN | FARMS | mmgMOS | MBEI | GCRMA |
| --- | --- | --- | --- | --- | --- | --- |
| *w* | 67.317% | 68.455% | 67.469% | 67.641% | 67.317% | 74.930% |
| WAD | 98.760% | 99.514% | 96.981% | 99.833% | 98.760% | 99.723% |
| AD | 99.501% | 99.775% | 97.422% | 99.734% | 99.501% | 99.762% |
| FC | 99.432% | 99.711% | 97.302% | 99.602% | 99.432% | 99.639% |
| RP | 99.403% | 99.769% | 98.975% | 99.708% | 99.403% | 99.808% |
| modT | 99.454% | 99.857% | 99.710% | 99.825% | 99.454% | 99.751% |
| samT | 99.619% | 99.878% | 99.715% | 99.870% | 99.619% | 99.765% |
| shrinkT | 99.380% | 99.842% | 33.623% | 99.838% | 99.380% | 99.754% |
| ibmT | 99.477% | 99.874% | 99.785% | 99.852% | 99.477% | 99.787% |

**Dataset 9**

**1.** http://www.ncbi.nlm.nih.gov/sites/entrez?Db=Pubmed&term=16617158[UID]

**2.** GSE2638 and GSE2639

**3.** 3 HMEC TNF-stimulated samples:

GSM50774-50776

**4.** 4 HUVEC TNF-stimulated samples:

GSM50781-50784

**5.** 16 probesets.

"205289_at", "206336_at", "209774_x_at", "203687_at", "210228_at",

"210229_s_at", "206618_at", "202688_at", "203917_at", "204748_at",

"221085_at", "221009_s_at", "204475_at", "211959_at", "206026_s_at",

"204580_at"

**6.** MAS5.0

**7.** Preprocessed data were obtained from a total of **14** samples in GSE2638 and GSE2639.

**8.**

| Method | PLIER | VSN | FARMS | mmgMOS | MBEI | GCRMA |
| --- | --- | --- | --- | --- | --- | --- |
| *w* | 52.392% | 53.913% | 61.329% | 57.692% | 57.267% | 64.406% |
| WAD | 77.711% | 81.290% | 87.778% | 88.279% | 81.537% | 91.689% |
| AD | 77.156% | 85.456% | 89.092% | 83.486% | 83.342% | 92.732% |
| FC | 79.360% | 85.805% | 89.288% | 83.202% | 83.619% | 92.765% |
| RP | 87.121% | 85.849% | 88.459% | 84.981% | 84.482% | 92.466% |
| modT | 76.549% | 79.520% | 77.454% | 82.957% | 76.547% | 87.425% |
| samT | 76.504% | 73.601% | 77.612% | 84.371% | 76.148% | 87.327% |
| shrinkT | 75.898% | 77.906% | 23.215% | 84.493% | 76.520% | 88.474% |
| ibmT | 77.635% | 81.114% | 80.758% | 85.274% | 76.549% | 88.386% |

**Dataset 10**

**1.** http://www.ncbi.nlm.nih.gov/sites/entrez?Db=Pubmed&term=15381369[UID]

**2.** GSE3524

**3.** 16 OSCE:

GSM80460-80475

**4.** 4 normal:

GSM80476, GSM80477, GSM80520, GSM80521

**5.** 4 probesets.

"201105_at", "204475_at", "211597_s_at", "213240_s_at"

**6.** MAS5.0

**7.** Preprocessed data were obtained from a total of 20 samples in GSE3524.

**8.**

| Method | PLIER | VSN | FARMS | mmgMOS | MBEI | GCRMA |
| --- | --- | --- | --- | --- | --- | --- |
| *w* | 65.242% | 86.557% | 74.213% | 73.439% | 82.082% | 84.871% |
| WAD | 99.046% | 99.823% | 99.824% | 99.930% | 99.751% | 99.902% |
| AD | 99.687% | 99.586% | 99.431% | 99.837% | 99.430% | 99.843% |
| FC | 99.037% | 99.917% | 99.910% | 99.652% | 99.877% | 99.732% |
| RP | 99.268% | 99.764% | 99.794% | 99.926% | 99.671% | 99.898% |
| modT | 95.652% | 94.232% | 90.976% | 98.186% | 90.754% | 97.605% |
| samT | 96.447% | 95.854% | 91.425% | 98.041% | 92.377% | 97.792% |
| shrinkT | 96.079% | 94.766% | 27.783% | 98.763% | 91.542% | 97.959% |
| ibmT | 96.117% | 94.598% | 92.117% | 98.678% | 91.161% | 97.814% |

**Dataset 11**

**1.** http://www.ncbi.nlm.nih.gov/sites/entrez?Db=Pubmed&term=15268757[UID]

**2.** GSE3860

**3.** 9 HGPS:

GSM87553, GSM87751, GSM88282-88288

**4.** 9 control:

GSM88289-88297

**5.** 8 probesets.

"206201_s_at", "201858_s_at", "201596_x_at", "210809_s_at", "200887_s_at",

"218899_s_at", "210239_at", "205830_at"

**6.** MAS5.0

**7.** Preprocessed data were obtained from a total of 18 samples in GSE3860.

**8.**

| Method | PLIER | VSN | FARMS | mmgMOS | MBEI | GCRMA |
| --- | --- | --- | --- | --- | --- | --- |
| *w* | 61.733% | 64.308% | 66.359% | 65.094% | 67.640% | 74.720% |
| WAD | 99.749% | 98.966% | 98.441% | 99.722% | 97.873% | 99.351% |
| AD | 99.750% | 99.746% | 99.255% | 99.574% | 98.891% | 99.588% |
| FC | 99.665% | 99.732% | 99.228% | 99.312% | 98.838% | 99.528% |
| RP | 99.811% | 99.843% | 99.521% | 99.620% | 99.032% | 99.673% |
| modT | 99.652% | 99.550% | 99.293% | 98.925% | 98.916% | 98.608% |
| samT | 99.725% | 99.656% | 99.303% | 98.889% | 98.977% | 98.697% |
| shrinkT | 99.571% | 99.525% | 21.818% | 99.131% | 98.873% | 98.653% |
| ibmT | 99.666% | 99.590% | 99.347% | 99.098% | 98.933% | 98.747% |

**Dataset 12**

**1.** http://www.ncbi.nlm.nih.gov/sites/entrez?Db=Pubmed&term=17181634[UID]

**2.** GSE5667

**3.** 5 control:

GSM132623-132627

**4.** 6 NLAD:

GSM132628-132633

**5.** 3 probesets.

"206407_s_at", "211338_at", "212592_at"

**6.** MAS5.0

**7.** Preprocessed data were obtained from a total of **17** samples in GSE5667.

**8.**

| Method | PLIER | VSN | FARMS | mmgMOS | MBEI | GCRMA |
| --- | --- | --- | --- | --- | --- | --- |
| *w* | 34.062% | 29.939% | 25.730% | 32.967% | 31.085% | 47.337% |
| WAD | 76.273% | 73.299% | 71.462% | 93.737% | 66.502% | 75.497% |
| AD | 80.684% | 80.163% | 75.286% | 99.327% | 66.730% | 78.182% |
| FC | 68.335% | 79.035% | 75.284% | 99.618% | 66.875% | 77.935% |
| RP | 81.344% | 73.740% | 75.162% | 99.180% | 66.692% | 73.402% |
| modT | 80.679% | 82.893% | 77.937% | 98.480% | 66.680% | 85.259% |
| samT | 79.035% | 82.825% | 78.076% | 97.982% | 66.765% | 85.184% |
| shrinkT | 79.095% | 82.871% | 26.565% | 98.775% | 66.617% | 83.193% |
| ibmT | 80.577% | 84.042% | 78.688% | 98.968% | 66.743% | 86.920% |

**Dataset 13**

**1.** http://www.ncbi.nlm.nih.gov/sites/entrez?Db=Pubmed&term=17181634[UID]

**2.** GSE5667

**3.** 5 control:

GSM132623-132627

**4.** 6 LAD:

GSM132634-132639

**5.** 3 probesets.

"206407_s_at", "211338_at", "212592_at"

**6.** MAS5.0

**7.** Preprocessed data were obtained from a total of **17** samples in GSE5667.

**8.**

| Method | PLIER | VSN | FARMS | mmgMOS | MBEI | GCRMA |
| --- | --- | --- | --- | --- | --- | --- |
| *w* | 39.798% | 33.278% | 33.362% | 39.687% | 35.435% | 50.491% |
| WAD | 69.361% | 67.397% | 76.207% | 81.556% | 74.565% | 73.206% |
| AD | 70.723% | 74.047% | 81.849% | 93.214% | 81.481% | 75.820% |
| FC | 67.524% | 75.009% | 82.089% | 94.511% | 82.086% | 75.660% |
| RP | 85.206% | 71.571% | 79.922% | 95.859% | 74.249% | 70.513% |
| modT | 70.723% | 68.553% | 82.747% | 73.799% | 78.731% | 71.851% |
| samT | 69.608% | 68.381% | 82.950% | 78.410% | 77.988% | 71.360% |
| shrinkT | 69.572% | 68.369% | 24.551% | 75.352% | 78.374% | 71.894% |
| ibmT | 70.691% | 69.913% | 83.683% | 76.275% | 80.690% | 73.405% |

**Dataset 14**

**1.** http://www.ncbi.nlm.nih.gov/sites/entrez?Db=Pubmed&term=17405831[UID]

**2.** GSE6236

**3.** 14 AB:

GSM143572-143585

**4.** 14 CB:

GSM143586-143599

**5.** 7 probesets.

"219630_at", "203388_at", "207854_at", "205838_at", "211935_at",

"217845_x_at", "221896_s_at"

**6.** MAS5.0

**7.** Preprocessed data were obtained from a total of 28 samples in GSE6236.

**8.**

| Method | PLIER | VSN | FARMS | mmgMOS | MBEI | GCRMA |
| --- | --- | --- | --- | --- | --- | --- |
| *w* | 71.419% | 76.600% | 77.356% | 87.845% | 79.578% | 95.823% |
| WAD | 99.681% | 99.176% | 99.144% | 99.781% | 98.961% | 99.572% |
| AD | 99.596% | 99.595% | 99.458% | 99.803% | 99.292% | 99.707% |
| FC | 99.605% | 99.585% | 99.461% | 99.772% | 99.221% | 99.710% |
| RP | 99.722% | 99.661% | 99.495% | 99.861% | 99.280% | 99.691% |
| modT | 99.606% | 99.738% | 99.707% | 99.658% | 99.229% | 99.770% |
| samT | 99.615% | 99.741% | 99.707% | 99.717% | 99.320% | 99.769% |
| shrinkT | 99.601% | 99.732% | 5.586% | 99.701% | 99.331% | 99.770% |
| ibmT | 99.652% | 99.742% | 99.707% | 99.708% | 99.250% | 99.771% |

**Dataset 15**

**1.** http://www.ncbi.nlm.nih.gov/sites/entrez?Db=Pubmed&term=17699851[UID]

**2.** GSE6344

**3.** 10 normal samples:

GSM146778, GSM146780, GSM146782, GSM146784, GSM146786,

GSM146789, GSM146790, GSM146792, GSM146794, GSM146796

**4.** 10 tumor samples:

GSM146779, GSM146781, GSM146783, GSM146785, GSM146787,

GSM146788, GSM146791, GSM146793, GSM146795, GSM146797

**5.** 19 probesets.

"202035_s_at", "202036_s_at", "202037_s_at", "208711_s_at", "208712_at",

"202431_s_at", "201667_at", "218995_s_at", "210495_x_at", "211719_x_at",

"212464_s_at", "216442_x_at", "201426_s_at", "200665_s_at", "201666_at",

"210512_s_at", "210513_s_at", "211527_x_at", "212171_x_at"

**6.** MAS5.0

**7.** Preprocessed data were obtained from a total of 20 samples in GSE6344.

**8.**

| Method | PLIER | VSN | FARMS | mmgMOS | MBEI | GCRMA |
| --- | --- | --- | --- | --- | --- | --- |
| *w* | 87.155% | 89.461% | 89.318% | 84.809% | 87.269% | 88.660% |
| WAD | 99.373% | 99.076% | 99.167% | 99.135% | 99.191% | 99.319% |
| AD | 98.563% | 99.538% | 99.434% | 96.978% | 99.418% | 98.587% |
| FC | 98.452% | 99.544% | 99.455% | 96.744% | 99.430% | 98.460% |
| RP | 98.327% | 99.445% | 99.440% | 97.041% | 99.423% | 98.500% |
| modT | 99.161% | 97.679% | 97.606% | 97.371% | 97.472% | 98.204% |
| samT | 99.139% | 98.152% | 97.636% | 97.320% | 97.882% | 98.282% |
| shrinkT | 99.135% | 97.822% | 25.637% | 97.758% | 97.968% | 98.386% |
| ibmT | 99.218% | 97.955% | 97.684% | 97.748% | 97.735% | 98.306% |

**Dataset 16**

**1.** http://www.ncbi.nlm.nih.gov/sites/entrez?Db=Pubmed&term=16858420[UID]

**2.** GSE6710

**3.** 13 lesional skin samples:

GSM154768, GSM154770, GSM154772, GSM154774, GSM154776,

GSM154778, GSM154780, GSM154782, GSM154784, GSM154786,

GSM154788, GSM154790, GSM154792

**4.** 13 uninvolved skin samples:

GSM154769, GSM154771, GSM154773, GSM154775, GSM154777,

GSM154779, GSM154781, GSM154783, GSM154785, GSM154787,

GSM154789, GSM154791, GSM154793

**5.** 7 probesets.

"205990_s_at", "213425_at", "209800_at", "203697_at", "203698_s_at",

"219908_at", "208712_at"

**6.** MAS5.0

**7.** Preprocessed data were obtained from a total of 26 samples in GSE6710.

**8.**

| Method | PLIER | VSN | FARMS | mmgMOS | MBEI | GCRMA |
| --- | --- | --- | --- | --- | --- | --- |
| *w* | 65.024% | 62.630% | 59.055% | 71.379% | 58.792% | 72.663% |
| WAD | 98.095% | 94.192% | 96.119% | 97.551% | 92.976% | 98.601% |
| AD | 97.782% | 98.133% | 97.621% | 95.761% | 94.494% | 98.268% |
| FC | 97.985% | 98.109% | 97.591% | 95.471% | 94.433% | 97.929% |
| RP | 98.572% | 98.257% | 98.087% | 95.408% | 94.793% | 98.512% |
| modT | 97.732% | 98.189% | 98.518% | 97.797% | 94.679% | 97.037% |
| samT | 97.605% | 98.512% | 98.537% | 98.161% | 94.542% | 97.197% |
| shrinkT | 97.684% | 98.221% | 18.523% | 98.103% | 94.737% | 97.285% |
| ibmT | 97.736% | 98.399% | 98.580% | 97.896% | 94.637% | 97.272% |

**Dataset 17**

**1.** http://www.ncbi.nlm.nih.gov/sites/entrez?Db=Pubmed&term=17472435[UID]

**2.** GSE7146

**3.** 6 pre-clamp samples:

GSM172123, GSM172129, GSM172131, GSM172133, GSM172136,

GSM172140

**4.** 6 post-clamp samples:

GSM172125, GSM172130, GSM172132, GSM172134, GSM172138,

GSM172142

**5.** 6 probesets.

"213524_s_at", "203140_at", "215990_s_at", "201008_s_at", "201009_s_at",

"201010_s_at"

**6.** MAS5.0

**7.** Preprocessed data were obtained from a total of **12** samples in GSE7146.

**8.**

| Method | PLIER | VSN | FARMS | mmgMOS | MBEI | GCRMA |
| --- | --- | --- | --- | --- | --- | --- |
| *w* | 90.451% | 93.623% | 93.671% | 81.919% | 95.343% | 88.938% |
| WAD | 88.731% | 89.127% | 94.337% | 84.669% | 85.828% | 87.924% |
| AD | 87.872% | 87.208% | 93.438% | 84.746% | 84.429% | 86.546% |
| FC | 85.574% | 87.421% | 93.447% | 84.361% | 83.383% | 86.470% |
| RP | 84.539% | 85.602% | 91.799% | 82.845% | 87.504% | 84.295% |
| modT | 88.160% | 87.273% | 96.782% | 87.459% | 84.084% | 93.671% |
| samT | 88.106% | 87.272% | 96.878% | 85.510% | 84.223% | 93.457% |
| shrinkT | 88.155% | 87.138% | 16.739% | 85.270% | 84.071% | 91.385% |
| ibmT | 88.450% | 87.201% | 97.001% | 87.046% | 84.004% | 89.002% |

**Dataset 18**

**1.** http://www.ncbi.nlm.nih.gov/sites/entrez?Db=Pubmed&term=17517823[UID]

**2.** GSE7765

**3.** 3 Dioxin-treated samples:

GSM188014, GSM188018, GSM188022

**4.** 3 control (DMSO-treated) samples:

GSM188013, GSM188016, GSM188020

**5.** 13 probesets.

"205623_at", "202887_s_at", "218729_at", "204341_at", "209201_x_at",

"217028_at", "218559_s_at", "204285_s_at", "204286_s_at", "206115_at",

"203666_at", "209687_at", "217763_s_at"

**6.** MAS5.0

**7.** Preprocessed data were obtained from a total of **6** samples in GSE7765.

**8.**

| Method | PLIER | VSN | FARMS | mmgMOS | MBEI | GCRMA |
| --- | --- | --- | --- | --- | --- | --- |
| *w* | 63.295% | 62.729% | 61.317% | 66.675% | 60.950% | 66.808% |
| WAD | 99.520% | 97.047% | 98.349% | 99.578% | 97.654% | 96.336% |
| AD | 98.835% | 99.308% | 99.450% | 98.256% | 98.615% | 98.783% |
| FC | 98.885% | 99.226% | 99.430% | 97.465% | 98.246% | 98.798% |
| RP | 99.199% | 99.259% | 99.430% | 98.314% | 98.638% | 98.074% |
| modT | 98.367% | 99.100% | 98.204% | 97.585% | 99.072% | 98.025% |
| samT | 98.854% | 99.487% | 97.053% | 98.304% | 98.822% | 97.693% |
| shrinkT | 96.019% | 98.732% | 99.127% | 97.704% | 98.867% | 98.065% |
| ibmT | 99.385% | 99.240% | 98.097% | 99.226% | 98.841% | 99.084% |

**Dataset 19**

**1.** http://www.ncbi.nlm.nih.gov/sites/entrez?Db=Pubmed&term=15374838[UID]

**2.** GSE1650

**3.** 18 severe emphysema samples:

GSM28357, GSM28359, GSM28361, GSM28363-28369,

GSM28371, GSM28373, GSM28375, GSM28377, GSM28379,

GSM28381, GSM28383, GSM28385

**4.** 12 no/mild emphysema samples:

GSM28358, GSM28360, GSM28362, GSM28370, GSM28372,

GSM28374, GSM28376, GSM28378, GSM28380, GSM28382,

GSM28384, GSM28386

**5.** 8 probesets.

"211959_at", "204640_s_at", "219127_at", "201744_s_at", "202291_s_at",

"202995_s_at", "200872_at", "216652_s_at"

**6.** MAS5.0

**7.** Preprocessed data were obtained from a total of 30 samples in GSE1650.

**8.**

| Method | PLIER | VSN | FARMS | mmgMOS | MBEI | GCRMA |
| --- | --- | --- | --- | --- | --- | --- |
| *w* | 88.303% | 83.217% | 83.168% | 90.915% | 83.946% | 88.244% |
| WAD | 82.948% | 92.101% | 92.816% | 85.039% | 78.337% | 94.781% |
| AD | 73.543% | 92.805% | 93.388% | 69.474% | 77.611% | 94.126% |
| FC | 73.184% | 92.661% | 93.386% | 72.054% | 78.611% | 94.246% |
| RP | 78.187% | 92.098% | 93.159% | 61.796% | 85.691% | 93.295% |
| modT | 78.594% | 91.238% | 96.017% | 80.678% | 77.127% | 87.317% |
| samT | 79.563% | 92.177% | 96.447% | 81.925% | 77.410% | 89.574% |
| shrinkT | 79.124% | 91.159% | 38.903% | 79.922% | 77.449% | 89.263% |
| ibmT | 79.140% | 91.194% | 91.402% | 81.764% | 77.102% | 87.396% |

**Dataset 20**

**1.** http://www.ncbi.nlm.nih.gov/sites/entrez?Db=Pubmed&term=15598877[UID]

**2.** GSE1615

**3.** 4 untreated normal samples:

GSM27531-27534

**4.** 5 untreated PCOS samples:

GSM27536-27538, GSM27540, GSM27541

**5.** 8 probesets.

"205651_x_at", "202724_s_at", "218145_at", "203917_at", "205700_at",

"208740_at", "208741_at", "208742_s_at"

**6.** MAS5.0

**7.** Preprocessed data were obtained from a total of **13** samples in GSE1615.

**8.**

| Method | PLIER | VSN | FARMS | mmgMOS | MBEI | GCRMA |
| --- | --- | --- | --- | --- | --- | --- |
| *w* | 59.681% | 57.515% | 55.478% | 64.687% | 53.875% | 64.824% |
| WAD | 83.506% | 81.833% | 79.508% | 94.115% | 81.635% | 85.921% |
| AD | 80.779% | 86.292% | 80.634% | 88.785% | 87.654% | 88.439% |
| FC | 79.675% | 86.011% | 80.566% | 87.994% | 87.367% | 87.975% |
| RP | 79.166% | 82.979% | 83.641% | 84.979% | 87.596% | 89.043% |
| modT | 80.739% | 87.485% | 77.322% | 94.648% | 85.118% | 92.383% |
| samT | 80.236% | 88.071% | 77.939% | 94.424% | 86.227% | 92.320% |
| shrinkT | 80.138% | 88.244% | 37.459% | 94.034% | 85.876% | 91.995% |
| ibmT | 81.107% | 87.055% | 79.573% | 95.099% | 84.896% | 92.665% |

**Dataset 21**

**1.** http://www.ncbi.nlm.nih.gov/sites/entrez?Db=Pubmed&term=15598877[UID]

**2.** GSE1615

**3.** 4 untreated normal samples:

GSM27531-27534

**4.** 4 VPA-treated normal samples:

GSM27543, GSM27546, GSM27548, GSM27549

**5.** 8 probesets.

"205651_x_at", "202724_s_at", "218145_at", "203917_at", "205700_at",

"208740_at", "208741_at", "208742_s_at"

**6.** MAS5.0

**7.** Preprocessed data were obtained from a total of **13** samples in GSE1615.

**8.**

| Method | PLIER | VSN | FARMS | mmgMOS | MBEI | GCRMA |
| --- | --- | --- | --- | --- | --- | --- |
| *w* | 55.473% | 55.161% | 53.254% | 62.019% | 50.661% | 63.750% |
| WAD | 71.650% | 60.337% | 78.476% | 75.336% | 49.964% | 77.140% |
| AD | 69.302% | 60.498% | 79.043% | 73.198% | 50.686% | 79.652% |
| FC | 67.622% | 60.612% | 79.117% | 71.591% | 51.017% | 79.852% |
| RP | 57.493% | 64.931% | 82.311% | 74.354% | 51.516% | 81.250% |
| modT | 62.650% | 51.700% | 55.169% | 63.113% | 45.802% | 61.789% |
| samT | 62.954% | 52.836% | 56.053% | 69.029% | 47.850% | 56.625% |
| shrinkT | 58.204% | 52.459% | 30.696% | 66.941% | 45.400% | 71.630% |
| ibmT | 66.382% | 51.777% | 59.723% | 66.698% | 46.099% | 56.824% |

**Dataset 22**

**1.** http://www.ncbi.nlm.nih.gov/sites/entrez?Db=Pubmed&term=16089502[UID]

**2.** GSE2666

**3.** 4 bone marrow Rho^high^ samples:

GSM51401, GSM51403, GSM51405, GSM51407

**4.** 4 bone marrow Rho^low^ samples:

GSM51402, GSM51404, GSM51406, GSM51408

**5.** 5 probesets.

"209560_s_at", "209993_at", "206176_at", "204695_at", "202917_s_at"

**6.** MAS5.0

**7.** Preprocessed data were obtained from a total of **18** samples in GSE2666.

**8.**

| Method | PLIER | VSN | FARMS | mmgMOS | MBEI | GCRMA |
| --- | --- | --- | --- | --- | --- | --- |
| *w* | 62.884% | 72.555% | 67.990% | 67.096% | 65.403% | 73.241% |
| WAD | 57.896% | 92.258% | 91.277% | 97.678% | 94.944% | 95.074% |
| AD | 49.847% | 93.637% | 91.775% | 94.477% | 96.474% | 95.495% |
| FC | 46.817% | 93.638% | 91.774% | 93.507% | 96.311% | 94.970% |
| RP | 93.184% | 93.860% | 92.808% | 93.436% | 96.905% | 95.945% |
| modT | 46.995% | 85.175% | 71.501% | 86.835% | 84.833% | 78.594% |
| samT | 44.585% | 82.462% | 72.311% | 91.173% | 82.670% | 77.212% |
| shrinkT | 44.911% | 84.898% | 23.926% | 89.526% | 85.620% | 86.235% |
| ibmT | 49.372% | 86.083% | 75.405% | 91.424% | 86.296% | 78.094% |

**Dataset 23**

**1.** http://www.ncbi.nlm.nih.gov/sites/entrez?Db=Pubmed&term=16089502[UID]

**2.** GSE2666

**3.** 5 Umbilical cord blood Rho^high^ samples:

GSM51391, GSM51393, GSM51395, GSM51397, GSM51399

**4.** 5 Umbilical cord blood Rho^low^ samples:

GSM51392, GSM51394, GSM51396, GSM51398, GSM51400

**5.** 6 probesets.

"209560_s_at", "209994_s_at", "206176_at", "204695_at", "202917_s_at",

"214370_at"

**6.** MAS5.0

**7.** Preprocessed data were obtained from a total of **18** samples in GSE2666.

**8.**

| Method | PLIER | VSN | FARMS | mmgMOS | MBEI | GCRMA |
| --- | --- | --- | --- | --- | --- | --- |
| *w* | 50.059% | 58.247% | 53.958% | 54.641% | 49.355% | 66.121% |
| WAD | 94.249% | 90.746% | 89.111% | 96.843% | 87.331% | 91.600% |
| AD | 94.283% | 96.493% | 90.823% | 98.028% | 94.693% | 96.544% |
| FC | 97.307% | 96.319% | 90.642% | 94.953% | 94.500% | 95.521% |
| RP | 97.723% | 95.688% | 93.109% | 98.029% | 92.338% | 96.365% |
| modT | 94.516% | 96.886% | 93.710% | 94.336% | 94.703% | 93.919% |
| samT | 94.211% | 97.510% | 94.105% | 96.210% | 94.375% | 93.911% |
| shrinkT | 94.354% | 96.626% | 35.396% | 95.979% | 94.677% | 95.312% |
| ibmT | 94.964% | 97.534% | 94.690% | 96.100% | 95.623% | 94.337% |

**Dataset 24**

**1.** http://www.ncbi.nlm.nih.gov/sites/entrez?Db=Pubmed&term=17251300[UID]

**2.** GSE6740

**3.** 10 (A + C) CD4+ cells:

GSM154936, GSM155180, GSM155182, GSM155184, GSM155186,

GSM155189, GSM155192, GSM155200, GSM155202, GSM155204

**4.** 10 (N + L) CD4+ cells:

GSM155218, GSM155220, GSM155222, GSM155224, GSM155226,

GSM155228, GSM155230, GSM155233, GSM155235, GSM155237

**5.** 40 probesets.

"201292_at", "201649_at", "201890_at", "202086_at", "202095_s_at",

"202145_at", "202270_at", "202446_s_at", "202589_at", "202869_at",

"203153_at", "203554_x_at", "204211_x_at", "204415_at", "204439_at",

"204747_at", "204972_at", "204994_at", "205241_at", "205483_s_at",

"205569_at", "205660_at", "205692_s_at", "206133_at", "206486_at",

"206513_at", "206991_s_at", "208965_s_at", "209417_s_at", "209969_s_at",

"213629_x_at", "213797_at", "214453_s_at", "217933_s_at", "218400_at",

"218543_s_at", "218741_at", "219352_at", "220169_at", "222154_s_at"

**6.** MAS5.0

**7.** Preprocessed data were obtained from a total of **40** samples in GSE6740.

**8.**

| Method | PLIER | VSN | FARMS | mmgMOS | MBEI | GCRMA |
| --- | --- | --- | --- | --- | --- | --- |
| *w* | 70.344% | 72.910% | 71.759% | 71.968% | 70.419% | 71.625% |
| WAD | 99.462% | 98.546% | 96.511% | 99.652% | 97.705% | 96.503% |
| AD | 99.254% | 99.259% | 96.850% | 98.910% | 98.610% | 97.502% |
| FC | 99.431% | 99.174% | 96.825% | 98.425% | 98.322% | 97.389% |
| RP | 99.457% | 99.050% | 97.918% | 98.350% | 98.034% | 97.756% |
| modT | 99.692% | 99.713% | 99.351% | 99.777% | 98.014% | 98.080% |
| samT | 99.680% | 99.715% | 99.381% | 99.774% | 98.386% | 98.063% |
| shrinkT | 99.695% | 99.725% | 19.775% | 99.798% | 98.286% | 98.018% |
| ibmT | 99.701% | 99.713% | 99.413% | 99.823% | 97.999% | 98.205% |

**Dataset 25**

**1.** http://www.ncbi.nlm.nih.gov/sites/entrez?Db=Pubmed&term=17251300[UID]

**2.** GSE6740

**3.** 10 (A + C) CD8+ cells:

GSM155179, GSM155181, GSM155183, GSM155185, GSM155187,

GSM155190, GSM155195, GSM155201, GSM155203, GSM155206

**4.** 10 (N + L) CD8+ cells:

GSM155219, GSM155221, GSM155223, GSM155225, GSM155227,

GSM155229, GSM155232, GSM155234, GSM155236, GSM155238

**5.** 62 probesets.

"201292_at", "201649_at", "201890_at", "202086_at", "202095_s_at",

"202145_at", "202270_at", "202446_s_at", "202589_at", "202869_at",

"203153_at", "203554_x_at", "204211_x_at", "204415_at", "204439_at",

"204747_at", "204972_at", "204994_at", "205241_at", "205483_s_at",

"205569_at", "205660_at", "205692_s_at", "206133_at", "206486_at",

"206513_at", "206991_s_at", "208965_s_at", "209417_s_at", "209969_s_at",

"213629_x_at", "213797_at", "214453_s_at", "217933_s_at", "218400_at",

"218543_s_at", "218741_at", "219352_at", "220169_at", "222154_s_at",

"201930_at", "202760_s_at", "203236_s_at", "203658_at", "204070_at",

"204205_at", "205098_at", "205898_at", "206632_s_at", "212048_s_at",

"213060_s_at", "214617_at", "215691_x_at", "35254_at", "200731_s_at",

"209741_x_at", "212762_s_at", "212870_at", "213222_at", "218486_at",

"219526_at", "221763_at"

**6.** MAS5.0

**7.** Preprocessed data were obtained from a total of **40** samples in GSE6740.

**8.**

| Method | PLIER | VSN | FARMS | mmgMOS | MBEI | GCRMA |
| --- | --- | --- | --- | --- | --- | --- |
| *w* | 73.734% | 73.409% | 73.190% | 75.736% | 72.148% | 75.128% |
| WAD | 97.909% | 97.694% | 94.436% | 98.220% | 95.224% | 97.450% |
| AD | 96.861% | 98.663% | 94.986% | 95.422% | 96.419% | 98.204% |
| FC | 97.844% | 98.695% | 95.012% | 94.831% | 96.285% | 98.016% |
| RP | 98.000% | 98.716% | 96.395% | 94.414% | 96.490% | 98.336% |
| modT | 97.398% | 98.340% | 97.532% | 98.363% | 96.229% | 98.173% |
| samT | 97.400% | 98.434% | 97.608% | 98.402% | 96.256% | 98.253% |
| shrinkT | 97.430% | 98.533% | 23.117% | 98.202% | 96.441% | 98.325% |
| ibmT | 97.505% | 98.310% | 97.824% | 98.459% | 96.241% | 98.370% |

**Dataset 26**

**1.** http://www.ncbi.nlm.nih.gov/sites/entrez?Db=Pubmed&term=18058819[UID]

**2.** GSE9574

**3.** 15 reduction mammoplasty patient samples:

GSM241999-242013

**4.** 14 breast cancer patient samples:

GSM242014-242027

**5.** 5 probesets.

"209774_x_at", "209189_at", "202768_at", "208960_s_at", "208961_s_at"

**6.** MAS5.0

**7.** Preprocessed data were obtained from a total of 29 samples in GSE9574.

**8.**

| Method | PLIER | VSN | FARMS | mmgMOS | MBEI | GCRMA |
| --- | --- | --- | --- | --- | --- | --- |
| *w* | 80.554% | 82.159% | 84.529% | 73.639% | 79.734% | 81.778% |
| WAD | 74.918% | 99.277% | 98.283% | 98.151% | 96.392% | 99.086% |
| AD | 63.421% | 99.820% | 98.521% | 99.939% | 97.343% | 99.784% |
| FC | 62.247% | 99.775% | 98.483% | 99.817% | 94.944% | 99.564% |
| RP | 99.796% | 99.789% | 99.025% | 99.914% | 98.633% | 99.744% |
| modT | 54.614% | 99.660% | 99.466% | 99.503% | 94.230% | 99.773% |
| samT | 52.082% | 99.773% | 99.503% | 99.568% | 94.960% | 99.785% |
| shrinkT | 53.440% | 99.671% | 16.826% | 99.633% | 94.305% | 99.793% |
| ibmT | 55.709% | 99.717% | 99.512% | 99.600% | 94.363% | 99.803% |

**Dataset 27**

**1.** http://www.ncbi.nlm.nih.gov/sites/entrez?Db=Pubmed&term=16690749[UID]

**2.** GSE4917

**3.** 3 Dex-treated samples:

GSM109211, GSM109219, GSM109227

**4.** 3 Control (ethanol)-treated samples:

GSM109210, GSM109218, GSM109226

**5.** 5 probesets.

"210095_s_at", "212143_s_at", "205479_s_at", "211668_s_at", "204292_x_at"

**6.** RMA

**7.** Preprocessed data were obtained from a total of 24 samples in GSE4917.

**8.**

| Method | PLIER | VSN | FARMS | mmgMOS | MBEI | GCRMA |
| --- | --- | --- | --- | --- | --- | --- |
| *w* | 57.625% | 77.252% | 68.534% | 59.377% | 73.817% | 66.201% |
| WAD | 88.696% | 98.438% | 98.796% | 90.984% | 92.424% | 94.655% |
| AD | 92.439% | 98.857% | 99.047% | 96.614% | 90.025% | 95.737% |
| FC | 80.225% | 98.968% | 99.039% | 97.184% | 91.321% | 96.175% |
| RP | 98.902% | 99.258% | 99.424% | 93.452% | 85.685% | 96.200% |
| modT | 92.439% | 95.717% | 88.725% | 95.390% | 81.839% | 93.057% |
| samT | 86.346% | 97.160% | 89.046% | 96.173% | 80.522% | 92.302% |
| shrinkT | 84.793% | 92.801% | 21.090% | 95.004% | 78.559% | 93.999% |
| ibmT | 91.480% | 96.576% | 89.943% | 95.794% | 82.015% | 93.657% |

**Dataset 28**

**1.** http://www.ncbi.nlm.nih.gov/sites/entrez?Db=Pubmed&term=17854483[UID]

**2.** GSE7148

**3.** 7 high-lonely samples:

GSM172173-172179

**4.** 7 low-lonely samples:

GSM172180-172186

**5.** 10 probesets.

"202859_x_at", "211506_s_at", "201853_s_at", "39402_at", "205067_at",

"201694_s_at", "201693_s_at", "202768_at", "204748_at", "204415_at"

**6.** RMA

**7.** Preprocessed data were obtained from a total of 14 samples in GSE7148.

**8.**

| Method | PLIER | VSN | FARMS | mmgMOS | MBEI | GCRMA |
| --- | --- | --- | --- | --- | --- | --- |
| *w* | 58.884% | 63.199% | 63.231% | 57.266% | 59.849% | 63.202% |
| WAD | 95.596% | 96.300% | 98.741% | 98.708% | 97.479% | 98.509% |
| AD | 90.974% | 99.242% | 99.251% | 94.218% | 99.014% | 99.220% |
| FC | 95.172% | 99.437% | 99.372% | 96.586% | 99.275% | 99.432% |
| RP | 93.646% | 98.792% | 98.948% | 93.168% | 98.443% | 98.591% |
| modT | 74.827% | 84.630% | 76.844% | 78.209% | 83.150% | 86.234% |
| samT | 76.147% | 91.864% | 78.306% | 76.728% | 84.391% | 87.510% |
| shrinkT | 73.706% | 85.053% | 9.909% | 81.328% | 84.401% | 88.860% |
| ibmT | 79.173% | 86.938% | 77.382% | 82.069% | 84.587% | 87.580% |

**Dataset 29**

**1.** http://www.ncbi.nlm.nih.gov/sites/entrez?Db=Pubmed&term=15583081[UID]

**2.** GSE5967

**3.** 7 patient samples:

GSM138597-138603

**4.** 7 control samples:

GSM138611-138617

**5.** 6 probesets.

"211862_x_at", "210564_x_at", "208485_x_at", "211317_s_at", "214486_x_at",

"221477_s_at"

**6.** RMA

**7.** Preprocessed data were obtained from a total of **21** samples in GSE5967.

**8.**

| Method | PLIER | VSN | FARMS | mmgMOS | MBEI | GCRMA |
| --- | --- | --- | --- | --- | --- | --- |
| *w* | 94.476% | 94.851% | 95.031% | 95.416% | 94.084% | 93.415% |
| WAD | 97.996% | 99.036% | 94.865% | 99.440% | 99.042% | 99.732% |
| AD | 94.707% | 99.456% | 94.659% | 95.462% | 98.879% | 99.716% |
| FC | 93.665% | 99.489% | 94.710% | 95.569% | 98.787% | 99.571% |
| RP | 98.151% | 99.666% | 97.471% | 91.174% | 98.968% | 99.853% |
| modT | 95.600% | 99.332% | 99.391% | 98.618% | 98.782% | 99.826% |
| samT | 93.831% | 99.801% | 99.497% | 98.771% | 99.205% | 99.850% |
| shrinkT | 95.418% | 99.332% | 22.399% | 98.865% | 98.735% | 99.845% |
| ibmT | 94.782% | 99.202% | 84.390% | 99.189% | 98.458% | 99.894% |

**Dataset 30**

**1.** http://www.ncbi.nlm.nih.gov/sites/entrez?Db=Pubmed&term=17264171[UID]

**2.** GSE6011

**3.** 14 normal skeletal muscle samples:

GSM139501-139514

**4.** 23 DMD skeletal muscle samples:

GSM139515-139537

**5.** 10 probesets.

"202310_s_at", "211161_s_at", "205132_at", "205940_at", "206717_at",

"202965_s_at", "206633_at", "221355_at", "204948_s_at", "208782_at"

**6.** RMA

**7.** Preprocessed data were obtained from a total of 37 samples in GSE6011.

**8.**

| Method | PLIER | VSN | FARMS | mmgMOS | MBEI | GCRMA |
| --- | --- | --- | --- | --- | --- | --- |
| *w* | 86.413% | 90.826% | 87.446% | 86.540% | 86.624% | 88.976% |
| WAD | 90.032% | 97.958% | 95.796% | 97.675% | 97.380% | 97.018% |
| AD | 89.048% | 97.866% | 95.793% | 96.210% | 97.862% | 97.605% |
| FC | 92.324% | 97.786% | 95.772% | 95.375% | 97.777% | 97.570% |
| RP | 91.724% | 97.358% | 96.529% | 95.371% | 97.284% | 97.793% |
| modT | 90.917% | 98.202% | 98.739% | 96.200% | 98.736% | 97.216% |
| samT | 90.886% | 98.301% | 98.756% | 96.161% | 98.703% | 97.264% |
| shrinkT | 90.961% | 98.194% | 16.762% | 96.430% | 98.736% | 97.326% |
| ibmT | 90.898% | 98.152% | 98.812% | 96.401% | 98.724% | 97.273% |

**Dataset 31**

**1.** http://www.ncbi.nlm.nih.gov/sites/entrez?Db=Pubmed&term=17660348[UID]

**2.** GSE8562

**3.** 3 MCF7/c samples:

GSM212605-212607

**4.** 3 MCF7/XBP1 samples:

GSM212608-212610

**5.** 8 probesets.

"213419_at", "202224_at", "206569_at", "202431_s_at", "209803_s_at",

"217728_at", "208900_s_at", "200792_at"

**6.** RMA

**7.** Preprocessed data were obtained from a total of 6 samples in GSE8562.

**8.**

| Method | PLIER | VSN | FARMS | mmgMOS | MBEI | GCRMA |
| --- | --- | --- | --- | --- | --- | --- |
| *w* | 77.851% | 78.000% | 77.497% | 79.762% | 75.987% | 79.680% |
| WAD | 97.341% | 95.527% | 96.553% | 98.244% | 94.359% | 96.698% |
| AD | 92.117% | 98.369% | 97.892% | 89.604% | 97.556% | 97.977% |
| FC | 92.483% | 98.351% | 97.918% | 89.251% | 97.525% | 97.875% |
| RP | 93.637% | 98.632% | 98.319% | 88.677% | 97.505% | 98.111% |
| modT | 93.094% | 96.647% | 92.569% | 95.275% | 96.974% | 96.286% |
| samT | 93.055% | 96.462% | 92.668% | 95.800% | 96.530% | 96.351% |
| shrinkT | 92.440% | 95.980% | 33.939% | 96.017% | 96.309% | 96.526% |
| ibmT | 94.808% | 97.516% | 93.883% | 98.013% | 97.455% | 97.687% |

**Dataset 32**

**1.** http://www.ncbi.nlm.nih.gov/sites/entrez?Db=Pubmed&term=16620959[UID]

**2.** GSE1937

**3.** 4 TCPS samples:

GSM34736-34739

**4.** 4 DCOL samples:

GSM34744-34747

**5.** 12 probesets.

"210512_s_at", "210513_s_at", "211527_x_at", "212171_x_at", "209960_at",

"210755_at", "210998_s_at", "203665_at", "201147_s_at", "201148_s_at",

"201149_s_at", "201150_s_at"

**6.** RMA

**7.** Preprocessed data were obtained from a total of **12** samples in GSE1937.

**8.**

| Method | PLIER | VSN | FARMS | mmgMOS | MBEI | GCRMA |
| --- | --- | --- | --- | --- | --- | --- |
| *w* | 84.855% | 85.446% | 85.037% | 86.512% | 83.259% | 86.240% |
| WAD | 85.736% | 88.953% | 91.541% | 89.425% | 94.411% | 95.929% |
| AD | 79.772% | 85.958% | 90.849% | 77.686% | 92.149% | 93.446% |
| FC | 79.594% | 85.739% | 90.827% | 77.618% | 92.119% | 93.443% |
| RP | 84.001% | 92.366% | 92.699% | 77.114% | 92.125% | 94.354% |
| modT | 81.783% | 81.162% | 84.078% | 87.620% | 88.058% | 91.597% |
| samT | 80.447% | 77.076% | 84.373% | 82.037% | 87.467% | 91.620% |
| shrinkT | 82.764% | 78.285% | 25.504% | 87.427% | 86.252% | 92.040% |
| ibmT | 82.945% | 80.493% | 84.115% | 89.879% | 88.180% | 92.426% |

**Dataset 33**

**1.** http://www.ncbi.nlm.nih.gov/sites/entrez?Db=Pubmed&term=16358311[UID]

**2.** GSE1577

**3.** 9 T-LL samples:

GSM27065-27066, GSM27068-27069, GSM27071-27072,

GSM27074-27075, GSM27077

**4.** 10 T-ALL samples:

GSM27079, GSM27082-27083, GSM27085, GSM27087-27088,

GSM27091, GSM27093-27095

**5.** 9 probesets.

"201162_at", "201163_s_at", "201667_at", "210495_x_at", "211719_x_at",

"212464_s_at", "216442_x_at", "211997_x_at", "211998_at"

**6.** RMA

**7.** Preprocessed data were obtained from a total of **29** samples in GSE1577.

**8.**

| Method | PLIER | VSN | FARMS | mmgMOS | MBEI | GCRMA |
| --- | --- | --- | --- | --- | --- | --- |
| *w* | 81.030% | 84.176% | 89.017% | 65.798% | 83.126% | 81.547% |
| WAD | 99.369% | 99.226% | 99.632% | 99.755% | 99.628% | 99.740% |
| AD | 98.187% | 99.751% | 99.768% | 98.983% | 99.575% | 99.561% |
| FC | 97.969% | 99.699% | 99.681% | 98.619% | 99.529% | 99.313% |
| RP | 99.157% | 99.789% | 99.798% | 98.814% | 99.691% | 99.654% |
| modT | 98.590% | 98.991% | 99.384% | 99.147% | 99.016% | 99.626% |
| samT | 98.584% | 99.286% | 99.405% | 99.554% | 99.221% | 99.639% |
| shrinkT | 98.597% | 99.086% | 31.470% | 99.353% | 99.161% | 99.661% |
| ibmT | 98.691% | 99.145% | 99.416% | 99.346% | 99.135% | 99.656% |

**Dataset 34**

**1.** http://www.ncbi.nlm.nih.gov/sites/entrez?Db=Pubmed&term=15877233[UID]

**2.** GSE2240

**3.** 17 atrial appendages:

GSM40991-41005, GSM41008-41009

**4.** 5 control samples:

GSM41010-41014

**5.** 3 probesets.

"210239_at", "203997_at", "205508_at"

**6.** RMA

**7.** Preprocessed data were obtained from a total of **22** samples in GSE2240.

**8.**

| Method | PLIER | VSN | FARMS | mmgMOS | MBEI | GCRMA |
| --- | --- | --- | --- | --- | --- | --- |
| *w* | 73.063% | 71.330% | 80.856% | 70.358% | 70.688% | 65.778% |
| WAD | 86.445% | 95.223% | 91.523% | 81.556% | 97.672% | 95.528% |
| AD | 84.436% | 96.848% | 91.206% | 75.748% | 98.354% | 96.276% |
| FC | 88.247% | 96.911% | 91.182% | 76.822% | 98.378% | 96.445% |
| RP | 93.133% | 96.370% | 91.599% | 84.051% | 98.449% | 95.993% |
| modT | 87.442% | 95.703% | 84.969% | 78.775% | 96.525% | 86.848% |
| samT | 87.180% | 96.098% | 85.476% | 77.949% | 96.936% | 87.497% |
| shrinkT | 87.382% | 95.679% | 30.269% | 78.980% | 96.710% | 91.091% |
| ibmT | 87.675% | 95.794% | 85.465% | 79.428% | 96.528% | 85.572% |

**Dataset 35**

**1.** http://www.ncbi.nlm.nih.gov/sites/entrez?Db=Pubmed&term=15817885[UID]

**2.** GSE2240

**3.** 10 atrial fibrillation samples:

GSM40980-40989

**4.** 20 sinus rhythm samples:

GSM40990-41009

**5.** 9 probesets.

"214316_x_at", "204312_x_at", "202765_s_at", "208351_s_at", "210555_s_at",

"209239_at", "205026_at", "215253_s_at", "213453_x_at"

**6.** RMA

**7.** Preprocessed data were obtained from a total of **35** samples in GSE2240.

**8.**

| Method | PLIER | VSN | FARMS | mmgMOS | MBEI | GCRMA |
| --- | --- | --- | --- | --- | --- | --- |
| *w* | 62.456% | 67.716% | 66.006% | 68.775% | 67.238% | 68.230% |
| WAD | 80.982% | 92.155% | 78.563% | 78.538% | 74.257% | 89.067% |
| AD | 77.005% | 91.051% | 78.881% | 69.137% | 67.124% | 88.880% |
| FC | 75.427% | 90.740% | 78.849% | 67.036% | 66.102% | 88.649% |
| RP | 69.840% | 89.554% | 81.913% | 65.952% | 75.861% | 89.450% |
| modT | 85.716% | 92.569% | 93.515% | 82.891% | 70.093% | 88.809% |
| samT | 84.664% | 92.674% | 93.407% | 82.979% | 69.873% | 89.129% |
| shrinkT | 86.326% | 92.509% | 41.871% | 82.062% | 69.954% | 89.454% |
| ibmT | 85.915% | 92.227% | 84.822% | 82.342% | 70.169% | 88.795% |

**Dataset 36**

**1.** http://www.ncbi.nlm.nih.gov/sites/entrez?Db=Pubmed&term=16797695[UID]

**2.** GSE2531

**3.** 3 JEG3 samples:

GSM48266-48268

**4.** 4 BeWo samples:

GSM48269-48272

**5.** 17 probesets.

"202910_s_at", "204271_s_at", "204273_at", "206701_x_at", "201910_at",

"201911_s_at", "201562_s_at", "201563_at", "203665_at", "203510_at",

"211599_x_at", "213807_x_at", "213816_s_at", "205322_s_at", "205323_s_at",

"201286_at", "201287_s_at"

**6.** RMA

**7.** Preprocessed data were obtained from a total of **7** samples in GSE2531.

**8.**

| Method | PLIER | VSN | FARMS | mmgMOS | MBEI | GCRMA |
| --- | --- | --- | --- | --- | --- | --- |
| *w* | 52.007% | 58.123% | 59.604% | 53.267% | 56.655% | 53.776% |
| WAD | 76.671% | 87.954% | 89.079% | 94.719% | 84.765% | 87.341% |
| AD | 76.439% | 90.766% | 90.950% | 94.732% | 87.561% | 91.226% |
| FC | 77.052% | 90.742% | 90.976% | 94.267% | 87.180% | 91.274% |
| RP | 91.554% | 91.338% | 91.622% | 95.247% | 88.157% | 91.642% |
| modT | 75.740% | 94.183% | 89.398% | 93.149% | 85.846% | 89.533% |
| samT | 75.881% | 94.741% | 89.258% | 94.681% | 86.015% | 89.464% |
| shrinkT | 74.952% | 94.168% | 38.079% | 94.164% | 85.138% | 89.791% |
| ibmT | 77.104% | 94.279% | 90.134% | 93.427% | 86.103% | 90.143% |

**Dataset 37**

**1.** http://www.ncbi.nlm.nih.gov/sites/entrez?Db=Pubmed&term=16894394[UID]

**2.** GSE5389

**3.**10 bipolar samples:

GSM123243-123252

**4.** 11 control samples:

GSM123253-123263

**5.** 6 probesets.

"202346_at", "201133_s_at", "201672_s_at", "204313_s_at", "212216_at",

"203998_s_at"

**6.** RMA

**7.** Preprocessed data were obtained from a total of 21 samples in GSE5389.

**8.**

| Method | PLIER | VSN | FARMS | mmgMOS | MBEI | GCRMA |
| --- | --- | --- | --- | --- | --- | --- |
| *w* | 82.630% | 80.742% | 80.403% | 85.320% | 75.967% | 85.891% |
| WAD | 98.250% | 96.638% | 92.477% | 88.537% | 92.981% | 97.787% |
| AD | 96.454% | 98.361% | 92.818% | 76.002% | 92.001% | 97.346% |
| FC | 96.960% | 98.388% | 92.811% | 63.901% | 90.046% | 96.086% |
| RP | 90.896% | 97.652% | 93.640% | 76.835% | 92.925% | 96.686% |
| modT | 97.423% | 98.792% | 95.822% | 83.477% | 89.898% | 97.142% |
| samT | 97.702% | 99.024% | 96.286% | 82.272% | 89.952% | 97.381% |
| shrinkT | 97.610% | 98.854% | 25.675% | 82.784% | 89.990% | 97.519% |
| ibmT | 97.470% | 98.723% | 96.100% | 83.654% | 89.578% | 97.533% |

**Dataset 38**

**1.** http://www.ncbi.nlm.nih.gov/sites/entrez?Db=Pubmed&term=17950572[UID]

**2.** GSE5390

**3.** 7 down syndrome samples:

GSM123264-123270

**4.** 8 control samples:

GSM123271-123278

**5.** 8 probesets.

"222162_s_at", "217867_x_at", "208370_s_at", "209033_s_at", "200677_at",

"209686_at", "203381_s_at", "212377_s_at"

**6.** RMA

**7.** Preprocessed data were obtained from a total of 15 samples in GSE5390.

**8.**

| Method | PLIER | VSN | FARMS | mmgMOS | MBEI | GCRMA |
| --- | --- | --- | --- | --- | --- | --- |
| *w* | 85.646% | 84.315% | 85.309% | 84.988% | 82.019% | 81.796% |
| WAD | 98.514% | 97.535% | 95.048% | 90.782% | 96.305% | 96.984% |
| AD | 96.517% | 98.091% | 94.614% | 79.497% | 97.207% | 97.121% |
| FC | 96.706% | 98.114% | 94.621% | 78.759% | 97.268% | 96.888% |
| RP | 94.218% | 97.577% | 95.182% | 83.392% | 97.230% | 96.695% |
| modT | 97.784% | 98.473% | 96.022% | 86.521% | 97.086% | 93.827% |
| samT | 97.643% | 98.447% | 96.375% | 86.536% | 97.017% | 95.212% |
| shrinkT | 97.738% | 98.565% | 20.189% | 85.790% | 97.632% | 97.665% |
| ibmT | 97.689% | 98.523% | 94.915% | 87.669% | 96.989% | 93.802% |
