# Supplementary figures and images for: Ranking differentially expressed genes from Affymetrix gene expression data: methods with reproducibility, sensitivity, and specificity
Source: Algorithms Mol Biol. 2009 Apr 22;4:7. doi: 10.1186/1748-7188-4-7 (PMC2679019; doi:10.1186/1748-7188-4-7)

PLIER

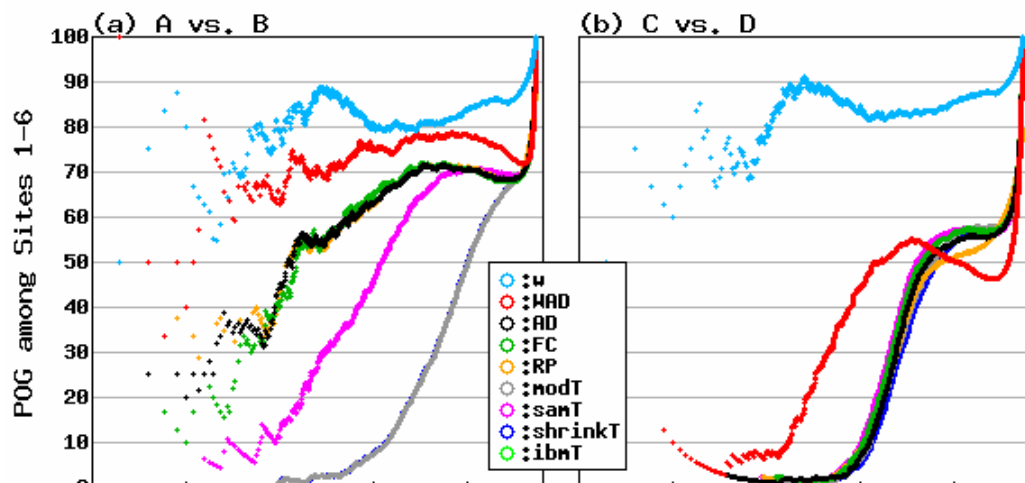

VSN

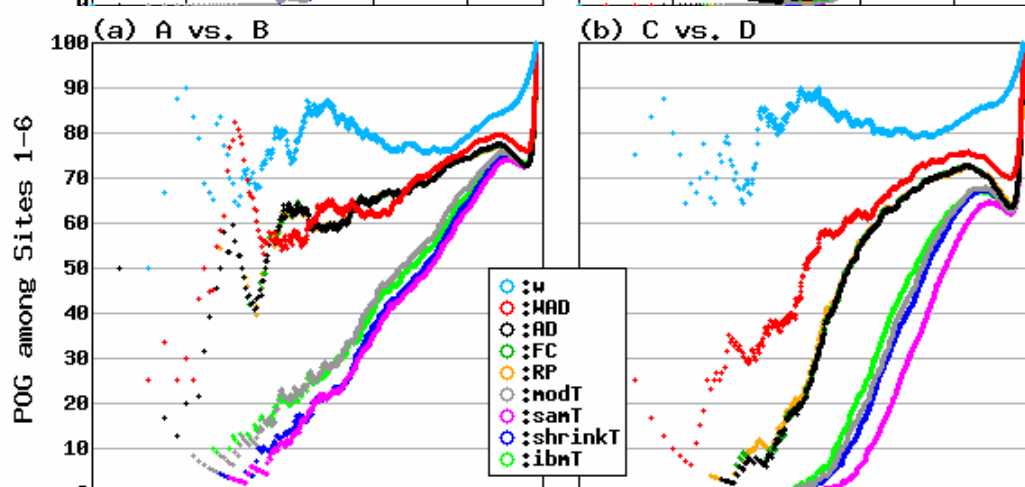

mmgMOS

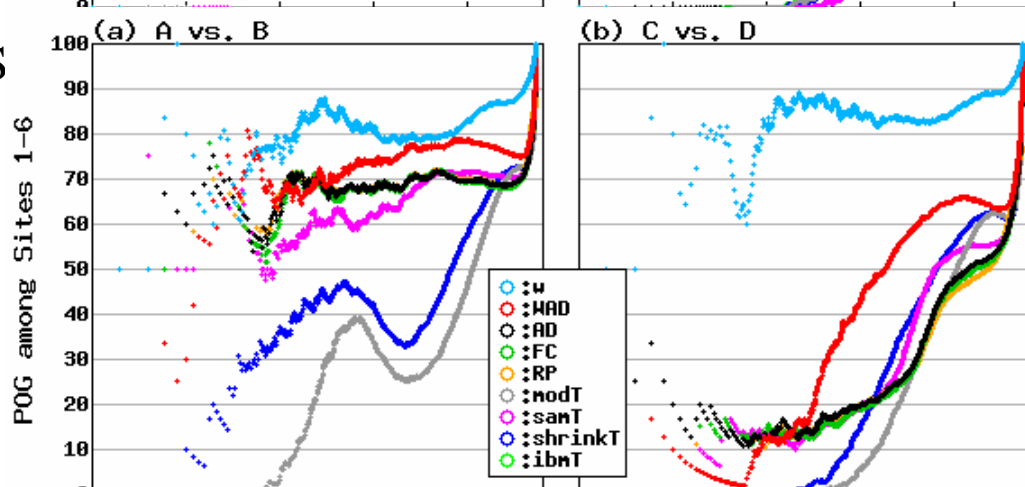

MBEI

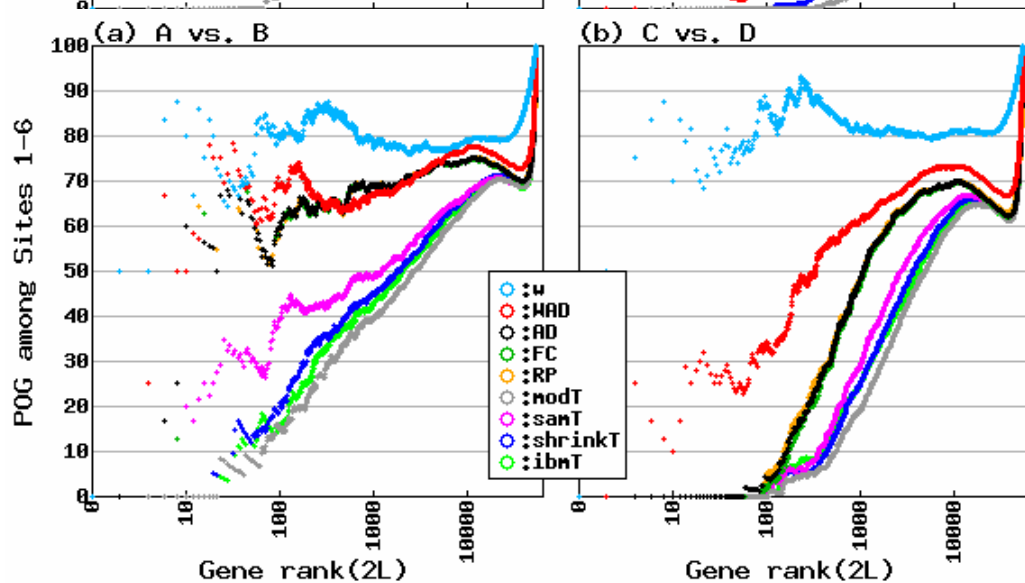

GCRMA

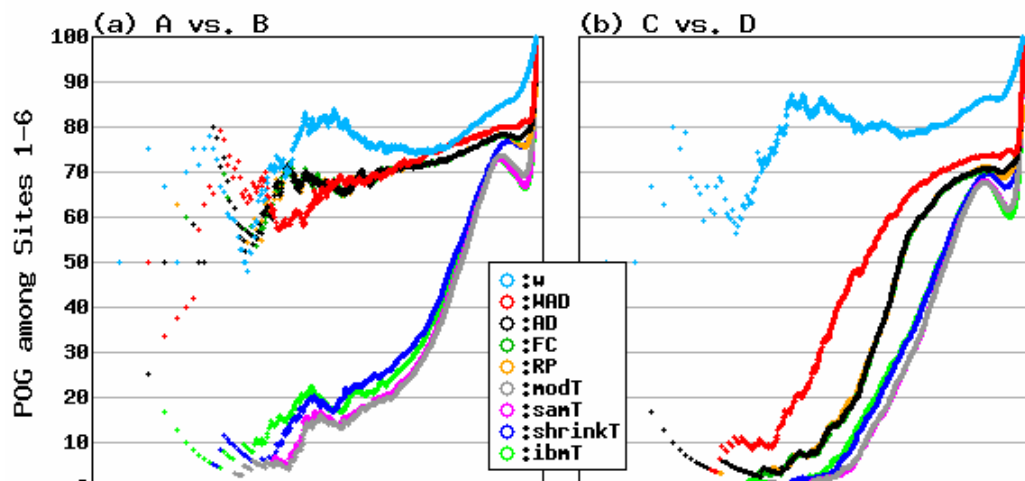

MAS

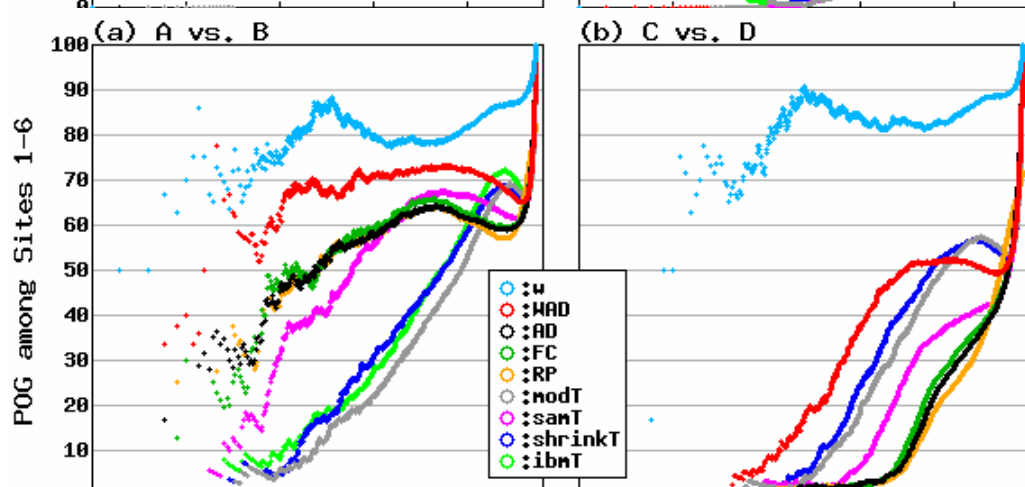

RMA

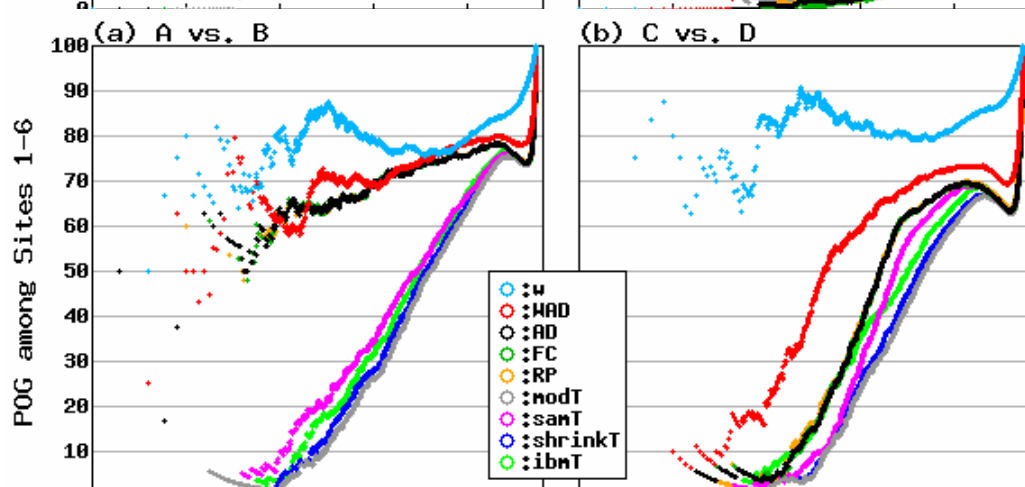

DFW

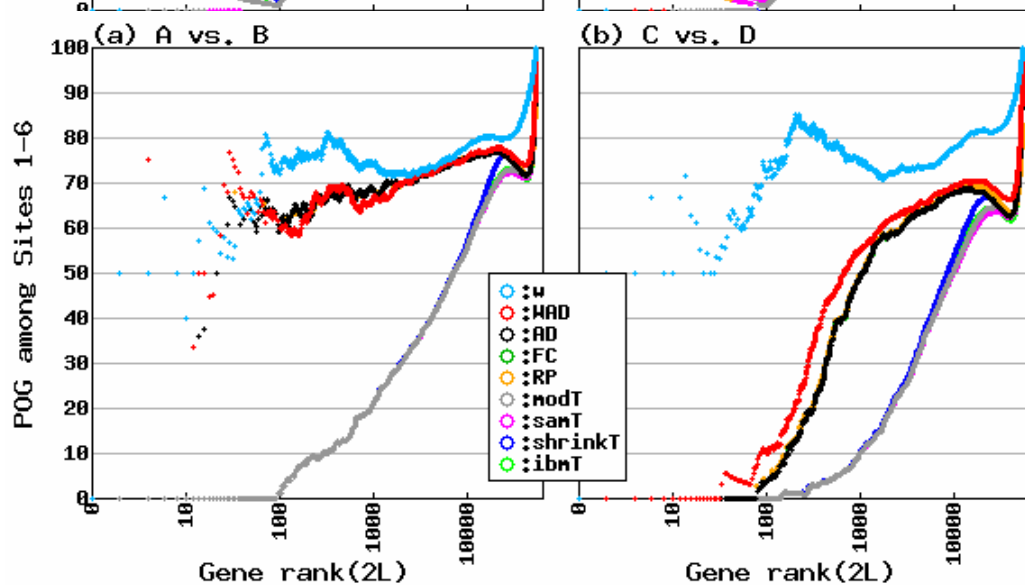

Supplement: Additional File 2 — POG values for PLIER-, VSN-, mmgMOS-, MBEI-, GCRMA-, MAS-, RMA-, and DFW-preprocessed data. Legends are the same as given in Figure 1. [file 1748-7188-4-7-S2.pdf]
